# Prone-Position Ventilation in a Pregnant Woman with Severe COVID-19 Infection Associated with Acute Respiratory Distress Syndrome

**Published:** 2020-11

**Authors:** Guitti Pourdowlat, Amir Mikaeilvand, Mitra Eftekhariyazdi, Mohammad Nematshahi, Masoud Ebrahimi, Asghar Kazemzadeh

**Affiliations:** 1 Chronic Respiratory Diseases Research Center, National Research Institute of Tuberculosis and Lung Disease (NRITLD), Shahid Beheshti University of Medical Sciences, Tehran, Iran,; 2 Department of Cardiology, Shahid Taleghani Teaching Hospital, Urmia University of Medical Sciences, Urmia, Iran,; 3 Department of Obstetrics and Gynecology, Vasei Hospital, Sabzevar University of Medical Sciences, Sabzevar, Iran,; 4 Department of Anesthesiology, Vasei Hospital, Sabzevar University of Medical Sciences, Sabzevar, Iran,; 5 Department of Internal Medicine, Infectious Diseases Ward, Vasei Hospital, Sabzevar University of Medical Sciences, Sabzevar, Iran,; 6 Department of Internal Medicine, Pulmonology and Respiratory Medicine Ward, Vasei Hospital, Sabzevar University of Medical Sciences, Sabzevar, Iran

**Keywords:** COVID-19, Prone-position ventilation, Pregnancy, Acute respiratory distress syndrome

## Abstract

A 25-year-old pregnant woman (gestational age: 24 weeks) presented with severe coronavirus disease-2019 (COVID-19) infection. Deterioration of her respiratory status resulted in her admission to the intensive care unit and mechanical ventilator support. Considering the lack of improvement in oxygen saturation, teleconsultation was performed, suggesting prone-position ventilation (PPV). Significant improvements were observed in oxygen saturation. The patient was extubated after five days of intermittent PPV and supine-position ventilation and was discharged 20 days after admission. Also, assessments revealed that the fetus was unharmed by the intervention. We suggest considering PPV for pregnant women with acute respiratory distress syndrome (ARDS).

## INTRODUCTION

The ongoing coronavirus disease-2019 (COVID-19) pandemic, caused by SARS-CoV-2, has given rise to many new challenges and difficulties, especially in vulnerable populations, such as the elderly and pregnant women. This report describes the unreported impact of prone-position ventilation (PPV) on refractory low oxygen saturation in a pregnant woman.

## CASE SUMMARY

**Figure 1.**
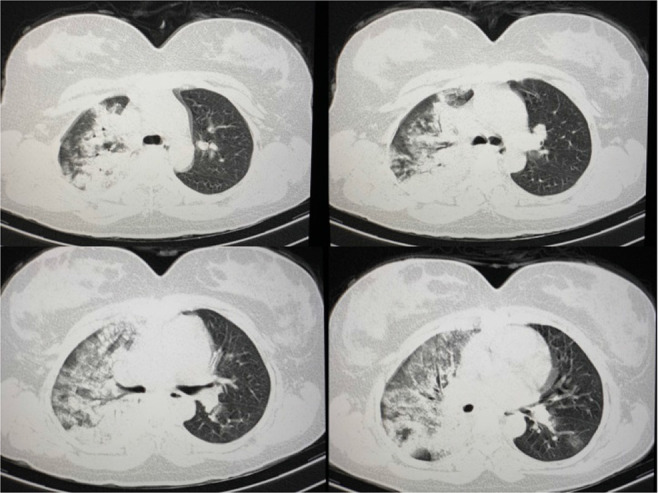
CT-scan imaging revealing severe pulmonary involvement prior to PPV

A febrile 25-year-old pregnant woman was admitted to our medical center. She lacked respiratory symptoms, and ultrasonography revealed a gestational age (GA) of 24 weeks. Based on the initial diagnosis of urinary tract infection, amikacin was administered for the patient. Deterioration of her clinical condition and development of dyspnea on the third day prompted further investigations. Computed tomography (CT) imaging revealed peripherally distributed ground-glass opacities. Therefore, diagnosis of COVID-19 infection was suspected (Figure 1).

After collecting nasopharyngeal samples for confirmatory tests, the patient was transferred to the COVID-19 intensive care unit (ICU). Accordingly, Kaletra and hydroxychloroquine were prescribed, and amikacin was replaced with meropenem and vancomycin. The result of the initial reverse transcription-polymerase chain reaction (RT-PCR) assay was negative. Deterioration of dyspnea, elevated respiratory rate (>40/min), and O_2_ saturation <85% with non-invasive ventilation led the medical team to sedate and intubate the patient for mechanical ventilator support on the fifth day. The result of COVID-19 RNA RT-PCR assay of secretions, obtained from the tracheal tube, was positive, confirming the diagnosis of this infection.

On the sixth day, despite mechanical ventilation with 100% FiO_2_ and constant sedation, the patient’s O_2_ saturation level remained below 83%. Teleconsultation was performed with other specialists from other centers, and PPV was suggested. After 30 minutes of PPV, O_2_ saturation increased to 92% and remained stable. Reduction of positive end expiratory pressure (PEEP) and FiO_2_ had no impacts on O_2_ saturation, as it remained above 93%. Also, the fetus remained unharmed, as evidenced by normal and reactive fetal heart monitoring (FHR). However, reversion to the supine position was associated with an almost immediate drop in O_2_ saturation to 90%, even at 100% FiO_2_.

**Figure 2.**
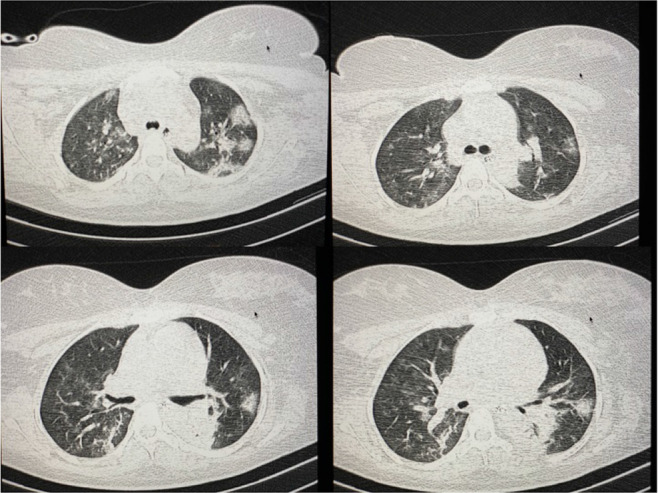
Improvement of pulmonary parenchymal involvement after PPV

On the following days, the patient was alternately ventilated in the prone (16 h/d) and supine (8 h/d) positions. On the tenth day, O_2_ saturation was stabilized at 93% in the supine position. On the 12th day, the results of clinical and paraclinical tests began to improve. Also, ultrasonography of the fetus confirmed normal fetal development and absence of discernible abnormalities. To reassure the mother of fetal health, the fetal heart sounds were played back for her on the 13th day. Also, sedatives were reduced, and the ventilator mode was switched to continuous positive airway pressure (CPAP). The control CT imaging revealed improvements within the lung tissue (Figure 2). The patient was gradually weaned from ventilation between days 13 and 16 to ensure the stability of O_2_ saturation level, which remained above 93%.

On the 16^th^ day, the patient was fully extubated and placed under non-invasive ventilation (NIV) while maintaining O_2_ saturation >94%. On day 20, she was discharged after showing sufficient recovery in both clinical and paraclinical parameters, with an O_2_ saturation level >97% in room air pressure. Sedation was initiated with fentanyl and propofol, which were later replaced with atracurium and cisatracurium. Overall, the administered medications between days 6 and 12 were as follows: four doses of beta-interferon; methylprednisolone for three days; and low doses of furosemide for four days.

Normal cardiac function was established by consulting a cardiologist, based on transthoracic echocardiography. Neither the patient nor the fetus experienced any adverse effects during hospitalization, and the mother was discharged in a desirable condition after recovering from ventilation and infection. Hydroxychloroquine and azithromycin were prescribed for up to one week after discharge. The trend of changes in laboratory parameters is presented in Table 1. The administered medications are also summarized in Table 2.

**Table 1.** The evolution of laboratory parameters during hospitalization.

	**Day 1**	**Day 2**	**Day 5**	**Day 9**	**Day 12**	**Day 16**	**Day 20**
**White blood cells (/micro litter)**	7190	6600	13600	10900	8900	9400	7430
**Polymorphonuclear (%)**	80	77	93	92	89	90	78
**Lymphocyte (%)**	18	18	7	6	9	7	20
**Platelet (/micro litter)**	208000	202000	260000	148000	135000	190000	203000
**Hemoglobin (mg/dl)**	12.2	10.8	9.8	10.4	10.8	10	12.1
**Serum Creatinine(mg/dl)**	0.8	---	0.7	0.9	0.8	1.0	0.8
**Sodium (mEq/L)**	138	---	137	142	136	134	135
**Potassium (mEq/L)**	3.8	---	3.9	4.5	4.3	4.4	3.9
**ESR (erythrocyte sedimentation rate) (mm/h)**	12	---	---	---	---	---	12
**CRP (C-reactive protein)**	1+	2+	---	---	---	---	Negative
**Urine culture**	E. coli >100000	---	Negative	---	---	---	---
**Blood culture**	---	---	Negative	---	---	---	---
**LDH (lactate dehydrogenase) (unit/L)**	---	---	482	>1500	1076	580	485
**Liver function tests**	---	---	In normal range	3x NV	> 4x NV	< 3x NV	Near normal

Abbreviation: Normal Value=NV

**Table 2.** Time chart of medications used throughout hospitalization; V&M: vancomycin (1gr, twice daily) and meropenem (1gr,q8H); Amikacin (500mg, q8H); Kaletra: lopinavir/Ritanavir (II tablets, twice daily/discontinued after day 13 due to prolonged QT-interval [470ms]); Methylprednisolone (200mg, q8H); Interferon-beta (12,000,000 units, q.o.d); Lasix (2mg/h); Hydroxychloroquine (200mg, twice daily).

	**1**	**2**	**3**	**4**	**5**	**6**	**7**	**8**	**9**	**10**	**11**	**12**	**13**	**14**	**15**	**16**	**17**	**18**	**19**	**20**
**Hospitalization**																				
**Amikacin**																				
**V&M**																				
**Methylprednisolone**																				
**Interferon**																				
**Hydroxychloroquine**																				
**Kaletra**																				
**Lasix**																				

## DISCUSSION

Although reports regarding the impact of COVID-19 in pregnant women are sparse, normal and pregnant populations do not seem to be significantly different (1).

An estimated maternal mortality of 9% is expected in pregnant women with COVID-19 (2). There is a dilemma regarding ICU care and ventilation for severe cases, especially when a subset of pregnant women fail to recover (3). Although there is limited evidence supporting PPV in severe cases of COVID-19 infection and ARDS, this method has been suggested to improve the outcomes in pregnant women, without exposing the fetus to any risks (4,5).

A number of studies have previously reported that PPV is a safe approach, which may be even superior to supine-position ventilation in the third trimester of pregnancy, as it is associated with comparatively fewer respiratory and cardiovascular complications and risks to the developing fetus (6–10). However, there is a large knowledge gap regarding the efficacy of PPV for pregnant women with ARDS. Overall, the fight against the COVID-19 pandemic requires unified and undivided global efforts of medical personnel, as well as the general population. Also, the lockdown due to COVID-19 in many countries has increased the need for teleconsultation between medical centers and has provided an opportunity to gain valuable information through exposure to different thoughts and perspectives.

This report presented the case of a pregnant woman (GA: 24 weeks) with severe respiratory complications due to COVID-19 infection, associated with ARDS, who showed limited response to supine-position ventilation. Based on previous studies regarding the efficacy of PPV and its safety during pregnancy, we examined this method of ventilation in the present study and obtained positive outcomes. Overall, alternating PPV and supine-position ventilation, coupled with constant FHR monitoring, can be used until improvements in clinical and paraclinical parameters are achieved. Both FHR monitoring and ultrasonography indicated normal fetal development throughout the intervention. The patient was discharged without any complications.

Based on the present results, we strongly recommend PPV in pregnant women with COVID-19 infection, associated with ARDS. However, further extensive research is required to establish the advantages and disadvantages of PPV for both mother and fetus in this setting.
